# How Salty Is Too Salty? Designing Sodium Warning Label Policies to Identify High-Sodium Items on Restaurant Menus in the United States

**DOI:** 10.3390/nu16121797

**Published:** 2024-06-07

**Authors:** Alla Hill, DeAnna Nara, Sarah Sorscher, Aviva A. Musicus, Peter Lurie

**Affiliations:** 1 Center for Science in the Public Interest, 1250 I Street NW, Suite 500, Washington, DC 20005, USA; dnara@cspinet.org (D.N.); ssorscher@cspinet.org (S.S.); amusicus@cspinet.org (A.A.M.); plurie@cspinet.org (P.L.); 2 Department of Nutrition, Harvard T.H. Chan School of Public Health, 655 Huntington Avenue, Boston, MA 02115, USA

**Keywords:** sodium, menu labeling, nutrient warnings, warning labels, restaurants, MenuStat, nutrition information, food labeling

## Abstract

Two U.S. cities require chain restaurants to label menu items that exceed 100% of the Daily Value (DV) for sodium, informing consumers and potentially prompting restaurant reformulation. To inform policy design for other localities, this study determined the percentage of the top 91 U.S. chain restaurants’ menu items that would be labeled if a warning policy were established for menu items exceeding the thresholds of 20%, 33%, 50%, 65%, and 100% of the sodium DV for adults. We obtained U.S. chain restaurants’ nutrition information from the 2019 MenuStat database and calculated the percentage of items requiring sodium warning labels across the food and beverage categories at all the restaurants and at the full- and limited-service restaurants separately. In total, 19,038 items were included in the analyses. A warning label covering items with >20%, >33%, >50%, >65%, and >100% of the sodium DV resulted in expected coverage of 42%, 30%, 20%, 13%, and 5% of menu items at all the restaurants, respectively. At each threshold, the average percentage of items labeled per restaurant was higher among the full-service restaurants than the limited-service restaurants. These results suggest that restaurant warning policies with a threshold of 100% of the sodium DV per item would cover a minority of high-sodium menu items and that lower thresholds should be considered to help U.S. consumers reduce their sodium consumption.

## 1. Introduction

Overconsumption of sodium remains one of the major contributors to the high rate of diet-related chronic disease in the United States, with critical public health and economic implications. Excessive sodium intake increases the risk of hypertension [1], which in turn raises the risk of heart attack and stroke [2]. Therefore, in the U.S., individuals over the age of 14 are advised to lower their sodium intake to under 2300 milligrams (mg) per day, the sodium Daily Value (DV) for adults [3]. However, the average population intake of sodium for children aged 2–19 (2968 mg/day) and adults aged 20 and older (3463 mg/day) greatly exceeds the recommended intake levels [4]. Researchers have estimated that reducing adult Americans’ average sodium intake by 34% to 2300 mg/day over the span of 10 years would prevent 895,000 cardiovascular disease (CVD) events and 252,500 CVD-related deaths [5]. 

While some sodium is naturally occurring in many foods, it is estimated that more than 70% of sodium consumption can be attributed to packaged and restaurant foods, where sodium may be added in high amounts to enhance palatability and shelf life [1,6]. Restaurants alone account for 26% and 31% of the average daily sodium intake for children and adults, respectively [7]. However, consumers do not realize how much sodium they are consuming at restaurants; in one study, 90% of adults and 88% of adolescents underestimated the sodium levels in restaurant menu items by an average of 900–1000 mg per meal when the actual mean sodium content was 1300 mg per meal for adults and 1100 mg per meal for adolescents [8]. 

Sodium warnings in restaurants are one policy approach to educate U.S. consumers and reduce sodium in their diets. These disclosures appear on menus, menu boards or other locations that provide easily interpretable information to consumers about food items that contain excessive amounts of sodium. Sodium warnings may also encourage restaurants to reduce the sodium in their menu items to avoid having to label high-sodium items with a warning. Two U.S. cities, New York City and Philadelphia, have enacted policies requiring sodium warnings on chain restaurant menu items that contain more than 2300 mg, or 100% of the adult DV [9,10]. This threshold is substantially above the value of 460 mg, or 20% of the DV, used in the FDA’s general consumer nutrition advice to identify items that are “high” in sodium [3]. The New York City Department of Health and Mental Hygiene estimated in an unpublished study that their warnings would apply to roughly 10% of menu items sold in the city’s chain restaurants [11]. 

As other cities and states consider restaurant warning policies to reduce sodium consumption, some may explore adopting lower thresholds to help consumers identify more high-sodium items and to create an incentive for restaurants to reduce the sodium content still further. Despite increasing government interest in these policies, the extent to which restaurant menu items would qualify for sodium warnings at different thresholds has not been assessed. Thus, the primary aim of this research was to assess the percentage of all chain restaurant menu items that would be labeled if a warning label policy were established at various sodium thresholds, overall and by food/beverage category. The secondary aims of this study were to compare the warning label prevalence at different thresholds by restaurant type (full service and limited service); describe the distribution of sodium in menu items by food/beverage category across all the restaurants; and identify the highest-sodium items in each food category. 

## 2. Materials and Methods

### 2.1. Design and Data Source

To evaluate the distribution of sodium across menu items at chain restaurants, we used a cross-sectional study design using 2019 MenuStat data, the most recently available at the time of analysis. The MenuStat 2019 database contains nutrition information for food and beverage items at 91 of the highest-grossing chain restaurants in the United States [12]. The nutrition information in MenuStat is sourced directly from the posted information on restaurant websites. We categorized each restaurant in the dataset as either full service (“establishments primarily engaged in providing food services to patrons who order and are served while seated, i.e., waiter/waitress service, and pay after eating” [13]) or limited service (“establishments primarily engaged in providing food services, except snack and nonalcoholic beverage bars, where patrons generally order or select items and pay before eating” [14]). We referred to restaurants’ websites for information about the establishments and cross-referenced the categorization of U.S. chain restaurants from previous studies [15,16,17,18] that have categorized restaurants using similar categories for full service (e.g., sit down, full service) and limited service (e.g., fast food, fast casual) to determine the categorization for each restaurant in the sample.

MenuStat categorizes each menu item into one of twelve food categories (e.g., appetizers and sides, baked goods, beverages) and assigns a binary code that denotes whether restaurants have described items as kids’ menu items (labeled for kids, like “kids’ fries”), combination meals (described as including an entrée, side, and drink), limited-time offer or seasonal, regional (only sold in certain locations), or shareable (“the nutrition cannot be divided into a single serving, e.g., carafes, whole pies, quarts of ice cream, 2 L drinks”) [19]. The full sample before exclusions included 25,870 menu items across 12 food categories. We focused our analysis on menu items that would be eligible for a warning label under New York City’s law, which applies to any items meant to feed one person (including combination meals) above the threshold for sodium, in addition to any items meant to feed more than one person for which each individual serving is above the threshold for sodium [9]. We could not determine the sodium content per serving for the majority of multi-serving items because the serving size information was missing for 56% of items in the 2019 MenuStat database. We thus chose to be conservative and excluded all the multi-serving items from our analyses. MenuStat’s “shareable” category was not an exhaustive collection of all the multi-serving items, so in addition to excluding those items, we excluded all the menu items with names and descriptions containing keywords commonly used to denote multi-serving items (e.g., “shareable”, “family”; see Table A1 for a full list of search terms). We reviewed this list to ensure we were not accidentally excluding any single-serving items by cross-validating the item descriptions on restaurants’ websites. We also excluded items coded as a limited-time offer or regional. We additionally created new codes for MenuStat’s “kids’ meal items” and “combination meal items” so these items were separated into their own distinct categories for analysis. In total, the sample included 20,197 menu items from 91 different restaurants across 14 food categories. Items missing sodium data (n = 1159) were excluded from the analyses.

### 2.2. Statistical Analysis

For the primary analyses, we calculated the expected prevalence of sodium warning labels on restaurant menu items when the label applied to items with more than 20%, 33%, 50%, 65%, or 100% of the sodium DV for adults (>460 mg, >759 mg, >1150 mg, >1500 mg, and >2300 mg, respectively). We chose to evaluate the 20% DV threshold as it corresponds to the FDA’s guidance to consumers to identify foods that are high in sodium [3]. We selected the 33% DV threshold based on the assumption that an average restaurant customer will consume three meals a day, so a given meal might contain one-third of their total daily sodium. The 50% DV threshold represents half of a day’s worth of sodium based on current dietary guidance and the assumption that a customer may consume one higher-calorie, higher-sodium meal in a day, in addition to two other lower-calorie and -sodium meals and still consume no more than the recommended amount of sodium in a day. We selected the 65% DV threshold because it aligns with the adequate intake for sodium, which is the “recommended average daily nutrient intake level based on observed or experimentally determined approximations or estimates of nutrient intake by a group (or groups) of apparently healthy people who are assumed to be maintaining an adequate nutritional state” of sodium assumed to ensure nutritional adequacy based on estimates of intake by an apparently health group of people with adequate nutritional states [1,20]. Lastly, we selected the 100% DV threshold as it is the standard for existing sodium labeling policies in New York City and Philadelphia. We calculated the warning label prevalence at each of these thresholds across all the menu items and within each food/beverage category at all the restaurants. For the secondary analyses, we calculated the average prevalence of warning labels at each threshold for menu items by restaurant category: full service and limited service.

In additional secondary analyses, we calculated the median, interquartile range, mean, standard deviation, and range of the sodium content overall and within each food category across all the restaurants. We assessed the normality of the sodium distributions in each category by evaluating the skewness and kurtosis. We also used descriptive statistics to rank the 5 highest-sodium items in each food category across all the restaurants.

We conducted exploratory analyses to understand the percentage of all the menu items across all the restaurants and stratified by restaurant type that would be labeled at increments of 10% of the sodium DV starting from 10% DV and increasing up to 100% DV. We additionally calculated the percentage of each individual restaurant’s menu items that would be labeled at various sodium thresholds and the percentage of each restaurant’s menu items that would require a sodium warning label at the 100% DV sodium threshold by food category. All the data were analyzed in IBM^®^ SPSS^®^ Statistics for Windows version 29.0.1.0 (IBM Corp., Armonk, NY, USA) and the figures were created using Microsoft^®^ Excel^®^ for Windows 365 (Version 2405).

## 3. Results

The dataset consisted of 20,197 items, including 1159 items with missing sodium data and 19,038 menu items with sodium data from a total of 91 restaurants (n = 28 full service, n = 63 limited service). The distribution of sodium in the food categories was generally marked by positive skewness (more low values than in a normal distribution) and positive kurtosis (more peaked than in a normal distribution) (Table 1). Across all the restaurant menu items, the median sodium exceeded 1150 mg per item (>50% of the DV) in 3 of the 14 food categories: combo meals, sandwiches, and burgers. In every category except desserts, the maximum amount of sodium exceeded 100% of the DV. 

**Figure 1:**
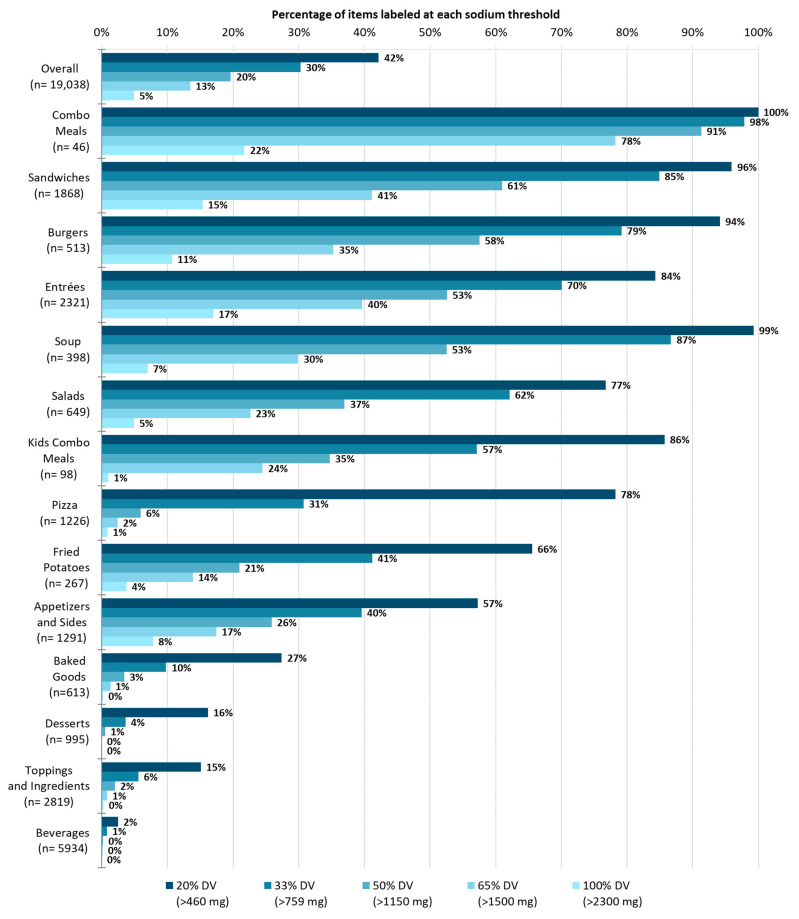
Expected prevalence of sodium warning labels on all the restaurant menu items overall and by food/beverage category when the label applies to items with >20%, >33%, >50%, >65%, or >100% of the Daily Value for sodium.

**Figure A1:**
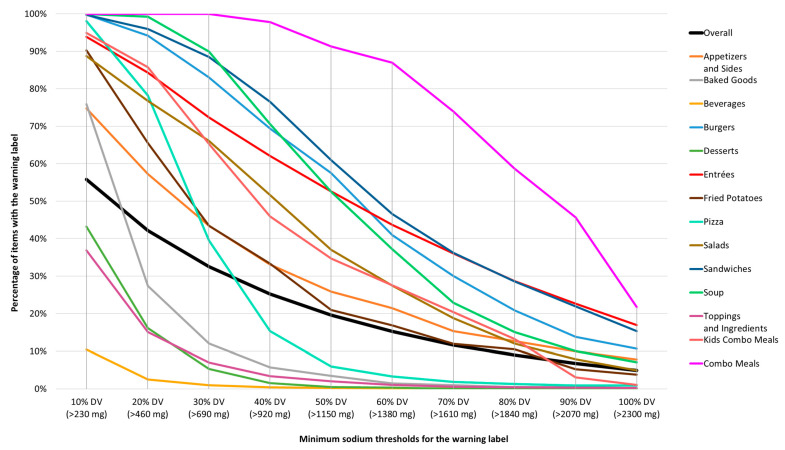
Expected prevalence of a sodium warning label on restaurant menu items when the label applies to items with 10–100% of the Daily Value for sodium.

Figure 1 represents the expected prevalence of the warning label within each food category at different thresholds. At the 20% DV threshold, 42% of all the items would be labeled (2–100% expected prevalence across categories), followed by 20% of items labeled at the 50% DV threshold (0–91%), and just 5% of items labeled at the 100% DV threshold (0–22%). Across the different thresholds, the combo meals, entrees, burgers, and soup categories generally had the highest expected prevalence of the label, whereas the beverages, toppings and ingredients, desserts, and baked goods categories generally had the lowest expected prevalence. Figure A1 shows the percentage of all the menu items across all the restaurants that would be labeled at increments of 10% of the sodium DV starting from 10% DV and increasing up to 100% DV. 

**Figure 2:**
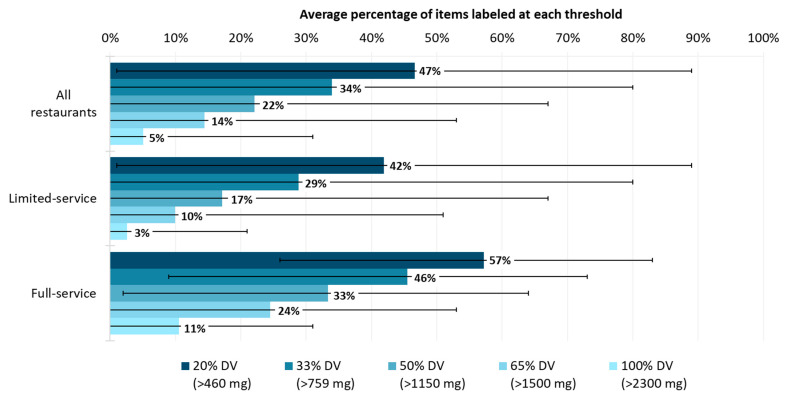
Percentage of menu items per restaurant that would require a sodium warning label when the label applies to items with >20%, >33%, >50%, >65%, or >100% of the Daily Value for sodium. Note: error bars represent the minimum and maximum average percentage of items labeled at each sodium threshold.

Figure 2 represents the average expected prevalence of items labeled at the various thresholds by restaurant type. On average, across all the restaurants, the expected prevalence of the label was 47% of items at the 20% DV threshold, while it decreased to 5% of items at the 100% DV threshold. We compared the expected prevalence of the label among items from all the restaurants with the expected prevalence for items from full- and limited-service restaurants. The average expected prevalence of the label was higher among the full-service restaurants than the limited-service restaurants at all the thresholds by 1.4-fold to 3.7-fold. At the 20% DV threshold, 42% of items from limited-service restaurants were labeled compared to 57% of items from full-service restaurants. And at the 100% DV threshold, just 3% and 11% of items were labeled at limited- and full-service restaurants, respectively.

**Figure A2:**
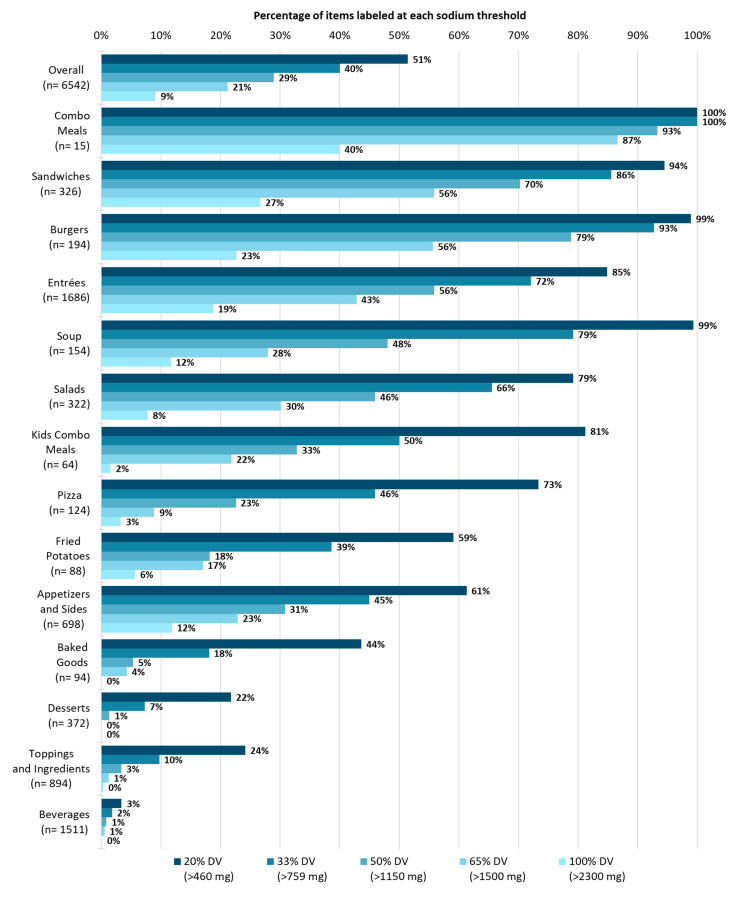
Expected prevalence of a sodium warning label on full-service restaurant menu items when the label applies to items with >20%, >33%, >50%, >65%, or >100% of the Daily Value for sodium.

**Figure A3:**
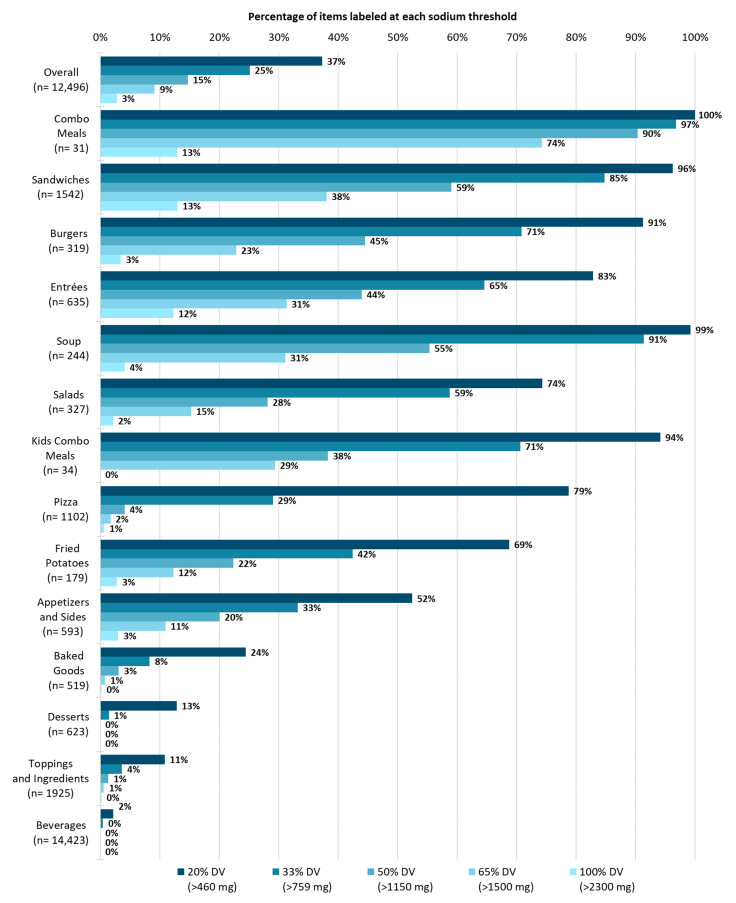
Expected prevalence of a sodium warning label on limited-service restaurant menu items when the label applies to items with >20%, >33%, >50%, >65%, or >100% of the Daily Value for sodium.

Figure A2 and Figure A3 show the percentage of items labeled at each threshold by food category within full-service and limited-service restaurants, respectively. At the 100% DV threshold, full-service restaurants had a higher percentage of items labeled overall and in most food categories when compared to limited-service restaurants, with the greatest differences observed in the percentage of items labeled in the combo meals, burgers, sandwiches, soups, and entrées categories. 

Table 2 shows the five highest-sodium items in each food category. The five highest-sodium items overall were the Chicken Noodle Soup Bowl from Fisch’s Big Boy (449% DV), the Whole Turkey Muffaletta from Jason’s Deli (383% DV), the Double Winder Boneless Wings with Buffalo Sauce from Famous Dave’s (343% DV), the Full Rack Baby Pack Pork Ribs from BJ’s Restaurant and Brewhouse (341% DV), and the Double Winder Boneless Wings with Devils Spit from Famous Dave’s (335% DV). For more information about individual restaurants, Table A2 contains the expected prevalence of sodium warning labels at each of the 91 restaurants at the various sodium thresholds, while Table A3 shows the percentage of menu items at each restaurant by food category that would require a sodium warning label when the label applies to items with >100% of the sodium DV. Of note, more than half of all the sandwiches offered at seven different restaurants would require a sodium warning at the 100% DV threshold. 

## 4. Discussion

This is the first evaluation, to the best of the authors’ knowledge, to examine the expected prevalence of sodium warnings on menu items across the top U.S. chain restaurants and stratified by restaurant type. We considered 5 potential sodium warning label thresholds (20%, 33%, 50%, 65%, and 100% of the adult DV) to estimate their impact on the percentage of labeled menu items across 14 food categories in a sample of 19,038 menu items from 91 chain restaurants in 2019. At the highest threshold, 100% of the sodium DV, or 2300 mg, only 5% of all the menu items would be expected to carry the warning label, and increasingly more items (compared to the change in the threshold) were expected to be labeled at the 65% threshold (13% of all the menu items), 50% threshold (20% of all the menu items), 33% threshold (30% of all the menu items), and 20% DV threshold (42% of all the menu items) due to the positively skewed distribution of sodium. Based on these findings, we expect that chain restaurant sodium warning policies using the warning threshold of 2300 mg, as in Philadelphia and New York City, are only covering a distinct minority of high-sodium menu items, thus leaving room to significantly strengthen these policies by applying reduced thresholds. 

Sodium warnings may be especially useful in full-service restaurants, as we found a higher expected prevalence of the label on items from full-service restaurants on average compared to limited-service restaurants at every sodium threshold. Furthermore, the five highest-sodium menu items in each food category largely came from full-service restaurants. Our study did not fully discern the extent to which the sodium content vs. a mix of offerings may play a role in the differences in the sodium content we observed at limited- vs. full-service restaurants. However, Figure A2 and Figure A3 show considerable differences in the sodium content of different offerings within the same categories between limited- vs. full-service restaurants. 

This study found that more than a day’s worth of salt can be hidden in menu items from food categories consumers likely would not suspect, including beverages, salads, and baked goods. This indicates that there is a clear need for policies like sodium warnings to help consumers understand the amount of sodium in the foods and beverages they order from restaurants. Our findings further provide data to inform threshold-setting for sodium warning policies. Sodium warnings are designed to be used by consumers to inform their intake around one meal. Considering that people in the U.S. average 5.7 eating occasions per day [21], consuming a menu item containing 100% of the DV would mean consumers would have to either avoid any additional dietary sodium for the remainder of the day to stay within the recommended limit, or substantially limit their sodium intake on adjacent days to maintain an average intake not exceeding the DV. Additionally, sodium added to foods during preparation is not the sole source of dietary sodium and some customers may add salt at the table after purchasing their food. Given the low percentage of labeled items with a 100% DV threshold and data suggesting such thresholds in New York City have not altered consumer behavior or restaurant reformulation [22,23], states and localities should consider setting sodium warning thresholds below the 100% sodium DV level to increase the effectiveness. However, a warning threshold set too low may lead to warning fatigue due to the high number of items that could be labeled (e.g., at the 20% DV threshold, the percentage of items labeled would be 42% overall and would exceed 50% in 10 of the 14 food categories), potentially desensitizing consumers to the warning [24]. However, reducing the threshold to 65% DV would leave the majority of menu items unlabeled in each food category except for one (combo meals), so consumers would still be likely to attend to the labels without ignoring them or becoming overwhelmed. 

No studies have tested the effects of lower-threshold sodium warnings on consumer behavior in a restaurant setting, but one study in a hospital cafeteria setting found that the introduction of sodium warnings at a 65% DV threshold was initially associated with a 6% decrease in sodium per item purchased [25]. However, the effects waned over 5 weeks. Importantly, half of the cafeteria’s customers were hospital staff who ate there frequently. This suggests that such labels have promise for reducing the sodium purchased and are most effective when salient, meaning that they may become less effective for people who habitually eat at the same restaurants. Furthermore, it is unclear how sodium warning labels at thresholds lower than 100% DV might spur restaurants to reformulate their menu items to contain less sodium, which is another important outcome of warning policies. It is possible that a warning policy requiring stepwise reductions in the sodium thresholds over time could increase the effectiveness by prolonging the salience. Future consumer testing should assess the potential for warning fatigue at various thresholds, while evaluations of enacted policies should examine the factors influencing restaurant reformulation. A gradual sodium reduction in restaurant foods may benefit restaurant owners and operators as well, as customers will have time to adjust to reduced-sodium menu items. Prior research has shown that up to a 50% sodium reduction did not affect consumers’ acceptance or liking of a food [26]. It follows that restaurant reformulation spurred by sodium warning policies may not even be noticed by customers, as they may be unable to detect taste changes in the dishes. Restaurateurs can explore other sodium-reduction strategies without compromising customer satisfaction and the taste quality of lower-sodium foods, such as the addition of herbs and spices [27,28].

This study has several limitations. First, our analyses were focused on the expected prevalence of warning labels among menu items and were not weighted based on actual consumption or sales data. As our restaurant sample and dataset did not include all the chain restaurants in the United States, we cannot generalize about the expected prevalence of a sodium warning label across smaller restaurants. We did examine the relationship between the number of outlets for a given chain and the prevalence of warnings at the 100% DV threshold and found that, after adjusting for the type of restaurant (full or limited service), there was no such relationship, suggesting that our findings may have relevance beyond these larger restaurants. A further limitation is that the restaurant nutrition information used in this study was from 2019 and menus may have changed. Furthermore, MenuStat relies on restaurants to post complete menus on their websites, and for 9 of the top 100 restaurants, data were not available. Lastly, some items intended to be shareable items may be in the dataset, although we attempted to exclude these based on coding for shareable items and a careful review of item descriptors indicating multi-serving items. It is also critical to point out that the 2300 mg daily sodium limit that policies have been based around—and that this study is based on—reflects the daily limit of sodium for individuals 14 years and older. Younger people need even less sodium per day, so policymakers should consider other ways to help parents choose foods with less sodium for their kids, like including sodium limits in kids’ meals [29].

## 5. Conclusions

In this study, a warning label applied to restaurant menu items with more than 2300 mg sodium resulted in a low expected sodium warning prevalence across 91 of the top 100 restaurant chains in the U.S. This is problematic, because menu items across all the examined food categories contained very high levels of sodium, and consumers are unlikely to be aware of this sodium content, especially for categories like beverages and salads. Therefore, policymakers considering restaurant warning label policies should consider a lower sodium threshold for sodium warning labels on restaurant menus.

## Figures and Tables

**Table 1:** Sodium (mg) in chain restaurant foods by food category.

	n	Median (Q1, Q3)	Min	Max	Mean (SD)	Skewness	Kurtosis
Combo meals ^a^	46	1990 (1560, 2280)	720	3390	1988 (572)	0.06	0.09
Sandwiches	1868	1330 (951, 1973)	50	8820	516 (816)	1.42	4.56
Burgers	513	1230 (850, 1720)	200	4950	1373 (745)	1.25	2.08
Entrées	2321	1220 (630, 1970)	0	7840	1417 (992)	1.14	1.82
Soup	398	1190 (870, 1570)	390	10,320	1365 (846)	4.35	34.26
Salads	649	940 (540, 1440)	5	3990	1033 (674)	0.79	0.58
Kids’ combo meals	98	845 (620, 1470)	20	2346	1018 (579)	0.51	−0.77
Pizza	1226	630 (490, 810)	60	6990	703 (463)	6.60	65.83
Fried Potatoes	267	620 (360, 1005)	30	4440	831 (674)	1.87	4.60
Appetizers and Sides	1291	590 (230, 1210)	0	7900	861 (929)	2.38	9.54
Baked Goods	613	360 (240, 480)	5	2840	413 (304)	2.67	12.00
Desserts	995	210 (100, 370)	0	1430	266 (224)	1.60	3.40
Toppings and Ingredients	2819	150 (40, 330)	0	3022	242 (306)	3.19	17.14
Beverages	5934	40 (10, 120)	0	2320	91 (148)	4.96	44.19

^a^ Category excludes kids’ menu combo meals. Q1, Q3: quartile 1, quartile 3. SD: standard deviation.

**Table 2:** Five highest-sodium items in each food category.

	Sodium (mg)	% Sodium DV	Item Name	Restaurant *^,†^
Combo Meals	3390	147%	Homestead Breakfast, High Calorie	Bob Evans ^†^
	3070	133%	Border Scramble Omelet, High Calorie	Bob Evans ^†^
	2820	123%	Homestead Breakfast, Low Calorie	Bob Evans ^†^
	2800	122%	Sirloin Steak and Farm Fresh Eggs, High Calorie	Bob Evans ^†^
	2790	121%	Honey BBQ Chicken Strip Sandwich Whatameal	Whataburger *
Sandwiches	8820	383%	Turkey Muffaletta, Whole	Jason’s Deli *
	5336	232%	Buffalo Chicken Grilled Cheese Sandwich	BJ’s Restaurant and Brewhouse ^†^
	4830	210%	Teriyaki Chicken Cheesesteak on Spinach Wrap	Jersey Mike’s Subs *
	4810	209%	Teriyaki Chicken Cheesesteak on Tomato Wrap	Jersey Mike’s Subs *
	4740	206%	The Big Bordurrito with Chicken	On the Border ^†^
Entrées	7840	341%	Baby Back Pork Ribs, Full Rack	BJ’s Restaurant and Brewhouse ^†^
	5960	259%	Shrimp Sampler	Joe’s Crab Shack ^†^
	5810	253%	10 Chicken Fingers with Teriyaki Sauce	Zaxby’s *
	5625	245%	Crispy Shrimp Platter	Ruby Tuesday ^†^
	5510	240%	10 Chicken Fingers with Insane Sauce	Zaxby’s *
Burgers	4950	215%	DD Burger	Hooters ^†^
	4283	186%	Hickory Brisket and Bacon Burger	BJ’s Restaurant and Brewhouse ^†^
	4060	177%	7 × 7 Steakburger	Steak N’ Shake *
	3860	168%	Twisted Texas Melt	Hooters ^†^
	3810	166%	Burger Sliders	Hooters ^†^
Soup	10,320	449%	Chicken Noodle Soup, Bowl	Frisch’s Big Boy *
	5160	224%	Chicken Noodle Soup, Cup	Frisch’s Big Boy *
	4937	215%	Chicken Tortilla in a Sourdough Loaf	BJ’s Restaurant and Brewhouse ^†^
	4683	204%	Clam Chowder in Sourdough Loaf	BJ’s Restaurant and Brewhouse ^†^
	4601	200%	Piranha Pale Ale Chili in Sourdough Loaf	BJ’s Restaurant and Brewhouse ^†^
Salads	3990	173%	Teriyaki Chicken Cheesesteak, In a Tub	Jersey Mike’s Subs *
	3370	147%	Chicken Taco Salad	Hooters ^†^
	3360	146%	Boneless Buffalo Chicken Salad	Chili’s ^†^
	3175	138%	Buffalo Steak Cheesesteak, In a Tub	Jersey Mike’s Subs *
	2990	130%	Grilled Salmon Superfood Salad	Bonefish Grill ^†^
Fried Potatoes	4440	193%	Loaded Waffle Fries	Friendly’s ^†^
	3350	146%	Chili Cheese Fries	Hooters ^†^
	3350	146%	VooDoo Fries with Fiery Ghost Pepper Sauce	Red Robin ^†^
	3140	137%	Lots a Tots	Hooters ^†^
	3070	133%	VooDoo Fries with Ranch Dressing	Red Robin ^†^
Appetizers and Sides	7900	343%	Boneless Wings with Buffalo Sauce, Double Winger	Famous Dave’s ^†^
	7700	335%	Boneless Wings with Devils Spit, Double Winger	Famous Dave’s ^†^
	7690	334%	Boneless Wings with Rich & Sassy, Double Winger	Famous Dave’s ^†^
	5970	260%	Beer Cheese and Pretzels	Hooters ^†^
	5490	239%	Pick 4 Sampler	Perkins ^†^
Pizza	6990	304%	Deep Deep Dish Specialty Pizza, 3 Meat Treat	Little Caesars *
	5720	249%	Deep Deep Dish Specialty Pizza, Hula Hawaiian Pizza with Ham	Little Caesars *
	5560	242%	Deep Deep Dish Specialty Pizza, Veggie	Little Caesars *
	5220	227%	Pepperoni, Hot N Ready Deep Deep Dish Pizza	Little Caesars *
	4660	203%	Pepperoni, Hot N Ready Classic	Little Caesars *
Baked Goods	2840	123%	Pepperoni Cheese Bread	Little Caesars *
	2200	96%	Italian Cheese Bread	Little Caesars *
	2090	91%	2 Biscuits	Perkins ^†^
	1820	79%	Hot N Ready Crazy Combo	Little Caesars *
	1780	77%	2 Biscuits, Breakfast	Steak N’ Shake *
Desserts	1430	62%	Chocolate Shack Attack	Joe’s Crab Shack ^†^
	1415	62%	Salted Caramel Pizookie	BJ’s Restaurant and Brewhouse ^†^
	1410	61%	The Great Wall of Chocolate	PF Chang’s ^†^
	1350	59%	Salted Caramel Cookie Skillet	Outback Steakhouse ^†^
	1230	53%	Cinnamon Sugar Doh Rings	Red Robin ^†^
Toppings and Ingredients	3022	131%	Hot and Spicy Buffalo	BJ’s Restaurant and Brewhouse ^†^
	2982	130%	BJs Original Wings	BJ’s Restaurant and Brewhouse ^†^
	2959	129%	EXXXXtra Hot Buffalo	BJ’s Restaurant and Brewhouse ^†^
	2930	127%	Hard Salami, for Whole Sandwich	Jason’s Deli *
	2756	120%	Queso, Bowl	Moe’s Southwest Grill *
Kids’ Combo Meals	2346	102%	Mini Burgers with Cheese, Kids	Ruby Tuesday ^†^
	2277	99%	Mac n Cheese Kids’ Meal	Bojangles *
	2152	94%	2 Piece Chicken Supremes Kids’ Meal	Bojangles *
	2060	90%	Corn Dog, Kids	Ruby Tuesday ^†^
	2010	87%	Crispy Chicken with Broccoli, Kids	California Pizza Kitchen ^†^
Beverages	2320	101%	El Nino Margarita	Chili’s ^†^
	2220	97%	Sicilian Prickly Pear Margarita	Carrabba’s Italian Grill ^†^
	2040	89%	Patron Margarita	Chili’s ^†^
	2040	89%	Presidente Margarita	Chili’s ^†^
	1830	80%	Ultimate Bloody Mary	California Pizza Kitchen ^†^

Note: Item names may not match names listed on restaurant menus due to MenuStat data entry protocol. DV: Daily Value. * Denotes limited-service restaurants, ^†^ Denotes full-service restaurants.

## Data Availability

MenuStat data are freely available online for public usage from www.menustat.org (accessed on 15 October 2021).

## Appendix A

**Table A1:** Search terms used to identify multi-serving items.

Search Terms	
Bottle	Pitcher
Bucket	Platter
Bundle	Quart
Family	Sack
Feast	Sampler
Feeds	Shareable
Gallon	To Share
Giant	Tray
Group	Whole Cake
Liter	Whole Pie
Party	
Picnic	

**Table A2:** Percentage of menu items for each restaurant that would require a sodium warning label when the label applies to items with >20%, >33%, >50%, >65%, or >100% of the Daily Value for sodium.

Restaurant Category	Restaurant Chain	Total # Items	% of Menu Items Labeled at Different Thresholds				
			20% DV	33% DV	50% DV	65% DV	100% DV
Full service	Applebee’s	258	45%	38%	28%	21%	14%
	BJ’s Restaurant and Brewhouse	430	53%	41%	29%	25%	11%
	Bob Evans	231	64%	54%	44%	32%	13%
	Bonefish Grill	94	48%	34%	20%	14%	5%
	California Pizza Kitchen	265	37%	28%	20%	11%	2%
	Carrabba’s Italian Grill	202	59%	46%	35%	27%	10%
	Chili’s	279	41%	32%	24%	20%	12%
	Denny’s	219	49%	35%	26%	18%	7%
	Famous Dave’s	222	80%	68%	52%	41%	15%
	Friendly’s	228	63%	54%	41%	26%	9%
	Golden Corral	720	26%	9%	2%	0%	0%
	Hooters	108	80%	69%	64%	53%	31%
	IHOP	327	46%	37%	28%	22%	8%
	Joe’s Crab Shack	191	49%	36%	26%	19%	14%
	LongHorn Steakhouse	182	49%	34%	17%	8%	1%
	O’Charley’s	158	78%	68%	56%	47%	24%
	Olive Garden	186	41%	31%	19%	12%	3%
	On the Border	167	69%	58%	47%	38%	17%
	Outback Steakhouse	95	82%	61%	37%	21%	5%
	Perkins	411	40%	28%	17%	12%	5%
	PF Chang’s	160	83%	73%	58%	46%	17%
	Red Lobster	250	52%	41%	30%	22%	8%
	Red Robin	400	45%	37%	27%	18%	6%
	Romano’s Macaroni Grill	95	80%	69%	54%	39%	15%
	Ruby Tuesday	101	73%	55%	44%	33%	15%
	TGI Friday’s	214	52%	45%	39%	30%	17%
	The Capital Grille	93	58%	44%	16%	10%	3%
	Yard House	256	57%	47%	34%	22%	9%
Limited service	Arby’s	82	67%	49%	38%	28%	6%
	Auntie Anne’s	153	22%	13%	7%	0%	0%
	Baskin Robbins	257	13%	1%	0%	0%	0%
	Bojangles	86	43%	27%	20%	12%	3%
	Boston Market	73	62%	38%	23%	10%	0%
	Burger King	180	41%	32%	15%	8%	1%
	Captain D’s	85	36%	20%	12%	9%	0%
	Carl’s Jr.	174	47%	37%	26%	15%	1%
	Casey’s General Store	183	38%	24%	16%	8%	0%
	Checker’s Drive-In/Rallys	79	54%	52%	33%	19%	0%
	Chick-Fil-A	127	46%	29%	16%	6%	1%
	Chipotle	69	26%	12%	1%	0%	0%
	Chuck E. Cheese	193	21%	11%	5%	1%	0%
	Church’s Chicken	141	30%	21%	14%	6%	2%
	Ci Ci’s Pizza	37	30%	19%	5%	5%	0%
	Culver’s	291	30%	19%	8%	5%	1%
	Dairy Queen	326	26%	19%	11%	7%	2%
	Del Taco	167	46%	32%	22%	12%	3%
	Dominos	381	20%	11%	3%	1%	0%
	Dunkin’ Donuts	482	10%	5%	2%	0%	0%
	Einstein Bros	145	57%	39%	23%	9%	1%
	El Pollo Loco	129	54%	45%	32%	22%	4%
	Firehouse Subs	826	6%	5%	4%	3%	2%
	Five Guys	31	29%	23%	13%	3%	3%
	Frisch’s Big Boy	121	59%	47%	30%	18%	7%
	Hardee’s	114	83%	71%	54%	29%	8%
	In-N-Out Burger	46	11%	7%	4%	0%	0%
	Jack in the Box	100	81%	64%	31%	16%	4%
	Jamba Juice	187	7%	1%	0%	0%	0%
	Jason’s Deli	344	43%	33%	24%	13%	4%
	Jersey Mike’s Subs	811	68%	59%	45%	35%	13%
	Jimmy John’s	46	67%	57%	48%	22%	2%
	KFC	185	18%	10%	6%	3%	1%
	Krispy Kreme	211	1%	0%	0%	0%	0%
	Krystal	72	43%	21%	10%	6%	1%
	Little Caesars	72	63%	39%	28%	19%	13%
	Long John Silver’s	86	23%	9%	5%	0%	0%
	Marco’s Pizza	248	25%	13%	7%	5%	3%
	McAlister’s Deli	185	71%	57%	35%	24%	5%
	McDonald’s	226	32%	26%	15%	6%	0%
	Moe’s Southwest Grill	125	17%	10%	5%	4%	2%
	Noodles and Company	80	76%	61%	38%	26%	4%
	Panda Express	104	27%	11%	1%	0%	0%
	Panera Bread	269	38%	28%	14%	7%	0%
	Papa John’s	804	51%	20%	2%	2%	1%
	Papa Murphy’s	111	77%	42%	8%	1%	0%
	Pizza Hut	157	47%	10%	2%	1%	0%
	Popeyes	79	53%	38%	29%	24%	3%
	Qdoba	63	32%	10%	3%	0%	0%
	Quiznos	117	79%	75%	48%	36%	21%
	Round Table Pizza	257	75%	25%	4%	2%	0%
	Sbarro	61	89%	80%	67%	51%	13%
	Sonic	425	24%	16%	8%	5%	1%
	Starbucks	371	9%	5%	2%	0%	0%
	Steak N’ Shake	445	25%	22%	14%	9%	3%
	Subway	121	56%	46%	26%	11%	3%
	Taco Bell	174	44%	31%	16%	2%	0%
	Tim Hortons	158	27%	19%	7%	1%	1%
	Wendy’s	111	38%	23%	13%	5%	0%
	Whataburger	218	60%	47%	33%	24%	5%
	White Castle	191	31%	14%	2%	1%	0%
	Wingstop	49	65%	45%	14%	4%	0%
	Zaxby’s	255	52%	42%	35%	27%	19%
		**Total # Items**	**Average% of Menu Items Labeled at Different Thresholds**				
			**20% DV**	**33% DV**	**50% DV**	**65% DV**	**100% DV**
All full-service restaurants		6542	57%	46%	33%	24%	11%
All limited-service restaurants		12,496	42%	29%	17%	10%	3%
All restaurants		19,038	47%	34%	22%	14%	5%

**Table A3:** Percentage of menu items by food category that would require a sodium warning label when the label applies to items with >100% of the Daily Value for sodium.

Restaurant Category	Restaurant Chain	% of Menu Items Labeled at the 100% DV Threshold *															
		Total # Items	Appetizers and Sides	Baked Goods	Beverages	Burgers	Desserts	Entrées	Fried Potatoes	Pizza	Salads	Sandwiches	Soup	Toppings and Ingredients	Kids’ combo Meals	Combo Meals	Total
Full service	Applebee’s	258	32%	0% *	0%	25% *	0%	46%	0% *	0% *	19% *	54%	0%	0%			14%
	BJ’s Restaurant and Brewhouse	430	3%	0% *	0%	73%	0%	21%	0%	0%	35%	37%	50%	3%	0% *		11%
	Bob Evans	231	0%	0% *	0%	0% *	0%	25%	0% *		16%	63%	0%	0%	0%	38%	13%
	Bonefish Grill	94	0%		0%	25% *	0%	6%	0% *		17%	14%	0% *	0%	0% *		5%
	California Pizza Kitchen	265	0%		0%		0%	6%		0%	0%	17%	0%	0%	0% *		2%
	Carrabba’s Italian Grill	202	10%		0%		0%	24%		0% *	0%	0%	33%	0%	0% *		10%
	Chili’s	279	0%	0% *	1%	17%	0% *	46%	0% *	0%	15%	54%	0%	0%			12%
	Denny’s	219	10%	0%	0%	0%	0%	14%	0%		0% *	27%	100% *	0%			7%
	Famous Dave’s	222	29%	0% *	0%	12%	0%	24%	0% *		0%	9%	0%	0%			15%
	Friendly’s	228	0%		0%	30%	0%	11%	25% *		0% *	11%	0% *	0% *	0%		9%
	Golden Corral	720	0%	0%	0%	0% *	0%	0%	0%	0%	0%	0%	0%	0%			0%
	Hooters	108	26%	0% *		67%	0% *	36%	40%		25%	45%	0% *	0%			31%
	IHOP	327	3%	0%	0%	0% *	0% *	16%	0% *		50%	29%	0% *	0%	0%		8%
	Joe’s Crab Shack	191	29%	0% *	0%		0%	32%	0% *	0% *	0% *	55%	0% *	0%	0% *		14%
	LongHorn Steakhouse	182	7%	0% *	0%	0%	0%	3%	0% *		0%	0%	0%	0%			1%
	O’Charley’s	158	7%	0% *		20%	0%	36%	0% *		0%	75%	40%	0%	0% *		24%
	Olive Garden	186	6%	0% *	0%		0%	11%	0% *	0% *	0% *	0%	0% *	0%	0% *		3%
	On the Border	167	30%		0%		0%	23%	0% *		0%	40%	0% *	0%	0%		17%
	Outback Steakhouse	95	0%			0%	0%	7%	0% *		0%	11%	33% *	0% *	0% *		5%
	Perkins	411	10%	0%	0%	0%	0%	15%	0%		0%	36%	0%	0%	0% *		5%
	PF Chang’s	160	5%		0%		0%	24%			0% *		57%	0% *			17%
	Red Lobster	250	13%	0% *	0%		0%	17%	0% *	0% *	0%	0%	0% *	0%			8%
	Red Robin	400	34%	0%	0%	3%	0%	16%	25%	0% *	0%	17%	0%	0%			6%
	Romano’s Macaroni Grill	95	0%	0% *	0%		0%	31%	0% *	0% *	5%	0% *		0% *	0% *		15%
	Ruby Tuesday	101	0%			13%	0%	27%	0% *	0% *	0% *	0% *	0% *	0%	33% *	50% *	15%
	TGI Friday’s	214	21%		0%	76%	0%	48%	0% *	0% *	8%	57%	0% *	0%			17%
	The Capital Grille	93	0%		0%	0% *	0%	8%	0% *		0%	0% *	17%				3%
	Yard House	256	12%	0% *	0%	0%	0%	31%	0% *	50%	3%	9%	0%	0%			9%
Limited service	Arby’s	82	0%	0% *	0%	0% *	0% *	0% *	0%		0% *	19%		0%			6%
	Auntie Anne’s	153		0%	0%					0% *		0%		0%			0%
	Baskin Robbins	257			0%		0%							0% *			0%
	Bojangles	86	0%	0% *	0%		0% *	20%	0% *		0% *	8%		0%	0% *		3%
	Boston Market	73	0%	0% *			0%	0%			0% *	0%	0%	0%			0%
	Burger King	180	0%	0% *	0%	0%	0%	13%	0%		0%	3%		0%	0% *		1%
	Captain D’s	85	0%	0% *	0%		0% *	0%	0% *		0%	0% *		0%			0%
	Carl’s Jr.	174	0%	0% *	0%	0%	0%	0%	0%		0%	8%		0%			1%
	Casey’s General Store	183	0% *	0%	0%	0% *	0%	0% *	0% *	0%	0% *	0%		0%			0%
	Checker’s Drive-In/Rallys	79	0%		0%	0%	0%	0% *	0%			0%					0%
	Chick-Fil-A	127	0%	0% *	0%		0% *	5%	0%		0%	0%	0% *	0%			1%
	Chipotle	69	0%		0% *									0%			0%
	Chuck E. Cheese	193	0%	0%			0%	0% *	0% *	0%		0% *		0%	0% *		0%
	Church’s Chicken	141	8%	0% *	0%	0% *	0% *	6%	0% *			0%		0%			2%
	Ci Ci’s Pizza	37	0% *	0% *			0% *	0% *		0%			0% *				0%
	Culver’s	291	8%	0% *	0%	0%	0%	14%	0% *		0%	0%	0% *	0%			1%
	Dairy Queen	326	0%	0% *	0%	0%	0%	39%	0%		0% *	0%		0% *			2%
	Del Taco	167	0% *	0% *	0%	0% *	0% *	20%	0%		0% *	4%					3%
	Dominos	381	0%	0%			0% *	0%		0%	0% *	0%		0%			0%
	Dunkin’ Donuts	482		0%	0%		0% *	0% *	0% *			0%		0%			0%
	Einstein Bros	145	0%	0%	0%		0% *	0% *		0% *	0% *	2%	0%	0%			1%
	El Pollo Loco	129	4%	0% *	0%			9%			0%	13%	0% *	0%			4%
	Firehouse Subs	826	0%		0%		0%				0%	39%	0% *	0%			2%
	Five Guys	31			0% *	50% *			0% *			0% *		0%			3%
	Frisch’s Big Boy	121	5%	0%	0%	0%	0%	11%	0% *		0% *	14%	33%	0%	0%		7%
	Hardee’s	114	0%	0% *	0% *	30%	0%	6%	0%		0% *	3%		0%			8%
	In-N-Out Burger	46			0%	0%			0% *								0%
	Jack in the Box	100	0%	0% *	0% *	6%	0% *	25%	0%		0%	3%		0%			4%
	Jamba Juice	187	0% *	0% *	0%			0%		0% *		0% *		0%			0%
	Jason’s Deli	344	0%	0%	0%	0% *	0%	14%		0% *	0%	14%	0%	1%			4%
	Jersey Mike’s Subs	811		0%	0%		0%	0% *			12%	27%	1%	0%			13%
	Jimmy John’s	46	0%		0%		0% *					3%		0% *			2%
	KFC	185	0%	0% *	0%		0%	4%	0% *		0% *	0%		0%			1%
	Krispy Kreme	211		0%	0%									0%			0%
	Krystal	72		0% *	0%	0%	0%	0%	0%			0%	100% *				1%
	Little Caesars	72	25%	17%	0% *					32%	0% *			0%			13%
	Long John Silver’s	86	0%	0% *	0%		0% *	0%	0% *			0% *	0% *	0%			0%
	Marco’s Pizza	248	0%	0% *	0%					0%	0%	39%		0%			3%
	McAlister’s Deli	185	0%		0%		0%	0%			0%	13%	0%	0% *	0% *		5%
	McDonald’s	226	0% *		0%	0%	0%	0%	0%		0%	0%		0%			0%
	Moe’s Southwest Grill	125	0%	0% *									50% *	1%			2%
	Noodles and Company	80	0% *	0% *			0% *	5%			0%		9%	0%			4%
	Panda Express	104	0%		0%		0% *	0%					0% *	0%			0%
	Panera Bread	269	0%	0%	0%		0%	0%			0%	2%	0%	0%			0%
	Papa John’s	804	6%	0%	0%		0% *			0%	0% *	40%		0%			1%
	Papa Murphy’s	111		0% *			0% *			0%	0%			0%			0%
	Pizza Hut	157	0%	0% *	0% *		0% *	0% *	0% *	0%				0%			0%
	Popeyes	79	0%	0% *	0%		0% *	6%	0% *			0%		11%			3%
	Qdoba	63	0% *				0% *					0%	0% *	0%			0%
	Quiznos	117			0% *		0% *				0% *	32%	0%	0%			21%
	Round Table Pizza	257	0%	0% *				0% *		0%	0% *	17%		0% *			0%
	Sbarro	61	11%					32%		0%	0%						13%
	Sonic	425	0%	0% *	0%	0%	0%	17%	9%			0%		0%			1%
	Starbucks	371	0% *	0%	0%		0%	0%			0%	0%		0% *			0%
	Steak N’ Shake	445	0%	0%	0%	3%	0% *	38%	17%		0%	0%	20%	0%	0%		3%
	Subway	121	0% *				0%			0% *	0%	7%	0%	0%			3%
	Taco Bell	174	0%	0%	0%			0%	0% *	0% *	0% *	0%		0%			0%
	Tim Hortons	158	0% *	0%	0%		0%	0%	0% *			4%	0%	0%			1%
	Wendy’s	111	0%	0% *	0%	0%	0% *	0%	0%		0%	0%	0% *				0%
	Whataburger	218	0% *	0% *	0%	4%	0%	25%	0% *		0%	4%		0%	0%	13%	5%
	White Castle	191	0%	0%	0%	0%	0% *	0% *	0%			0%					0%
	Wingstop	49	0%	0% *			0% *	0%	0% *					0% *			0%
	Zaxby’s	255	20%	0% *	0%		0% *	67%	0% *		8%	57%		4%	0% *		19%
		**Average % of Menu Items Labeled at the 100% DV Threshold**															
		**Total # Items**	**Appetizers and Sides**	**Baked Goods**	**Beverages**	**Burgers**	**Desserts**	**Entrées**	**Fried Potatoes**	**Pizza**	**Salads**	**Sandwiches**	**Soup**	**Toppings and Ingredients**	**Kids’ combo meals**	**Combo meals**	**Total**
All full-service restaurants		6542	10%	0%	0%	18%	0%	22%	4%	4%	7%	24%	12%	0%	2%	44%	11%
All limited-service restaurants		12,496	2%	0%	0%	4%	0%	8%	1%	2%	1%	8%	9%	0%	0%	13%	3%
All restaurants		19,038	5%	0%	0%	11%	0%	13%	2%	3%	3%	13%	11%	0%	1%	34%	5%

Note: Blank cells indicate there were no menu items within the food category for a particular restaurant. * Indicates the total number of items in a food category was less than 5.
